# Effect of fluorine on the photovoltaic properties of 2,1,3-benzothiadiazole-based alternating conjugated polymers by changing the position and number of fluorine atoms†

**DOI:** 10.1039/d4ra01104j

**Published:** 2024-04-11

**Authors:** Pengzhi Guo, Xuemei Gan, Sheng Guan, Peili Gao, Qian Wang, Furong Shi, Yuan Zhou, Chenglong Wang, Yangjun Xia

**Affiliations:** a National Engineering Research Center for Technology and Equipment of Green Coating, Lanzhou Jiaotong University Lanzhou 730070 China shxygpz@126.com +86-0931-495-6058; b Organic Semiconductor Materials and Applied Technology Research Center of Gansu Province, School of Material Science and Engineering, Lanzhou Jiaotong University Lanzhou 730070 China

## Abstract

Fluorination is one of the most effective ways to manipulate molecular packing, optical bandgap and molecular energy levels in organic semiconductor materials. In this work, different number of fluorine atoms was introduced into the acceptor moiety 2,2′-dithiophene linked 2,1,3-benzothiadiazole, utilizing the alkylthiophene modified dithieno[2,3-*d*:2′,3′-*d*′]benzo[1,2-*b*:4,5-*b*] (DTBDT) as the donor unit, three polymers: PDTBDT-0F-BTs, PDTBDT-2F-BTs and PDTBDT-6F-FBTs were synthesized. With the number of fluorine atoms in each repeat unit of polymers varying from 0 to 2 and then up to 6, PDTBDT-0F-BTs, PDTBDT-2F-BTs and PDTBDT-6F-FBTs exhibited gradually downshifted energy levels and improved dielectric constants (*ε*_r_) from 3.4 to 4.3 to 5.8, further successively increased charge transport mobilities. As a result, the power conversion efficiency (PCE) of the bulk heterojunction organic photovoltaic devices (BHJ-OPV) from the blend films of aforementioned polymers paired with PC_71_BM were gradually increased from 1.69 for PDTBDT-0F-BTs to 1.89 for PDTBDT-2F-BTs and then to 5.28 for PDTBDT-6F-FBTs. The results show that the continuous insertion of fluorine atoms into the repeating units of the benzothiadiazole conjugated polymer leads to the deepening of HOMO energy level, the increase of *ε*_r_ and the increase of charge mobility, which improve the efficiency of charge transfer and electron collection, thus improving the photovoltaic performance of BHJ-OPV.

## Abstract 2

Incorporation of fluorine atoms in conjugated polymers leads to an increase in charge mobility and dielectric constant, increasing the *P*_diss_ value and power conversion efficiency and open circuit voltage of polymer bulk heterojunction photovoltaic cells.

## Graphical abstract

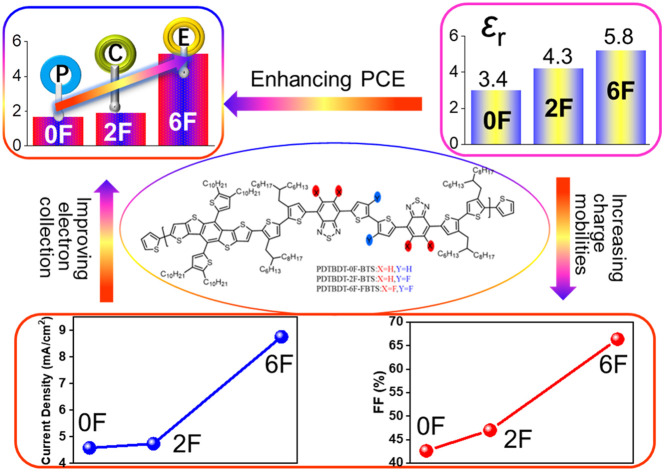

## 1. Introduction

In the past decades, bulk heterojunction organic photovoltaics devices (BHJ-OPVs) have garnered significant attention due to their flexibility, light-weight, large-area and low-cost.^1–3^ Recently, due to the development of the actively materials, the power conversion efficiencies (PCEs) of the single-junction OPVs have improved to over 19%.^4–7^ However, up to now, the PCEs of the OPVs are still lower than their inorganic rival photovoltaic devices. This disparity is primarily attributed to the low dielectric constant (*ε*_r_) of organic photovoltaics materials. Photoexcitation of OPVs leads to the formation of bound hole–electron pairs, termed Frenkel excitons.^8–10^ As in OPVs, the Coulomb interaction between the opposite electric charge carriers, which can be expressed as 
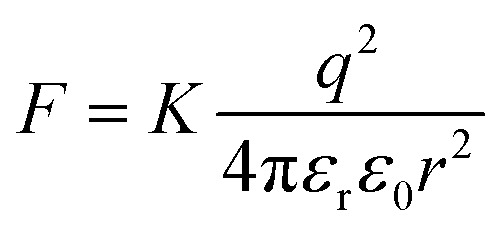
 are always need to be overcame in order to improve charge carrier generation, transporting and collection. The low *ε*_r_ for organic semiconductors has been considered as a major contributor to recombination losses in OPVs, including monomolecular and bimolecular recombination.^11,12^ Therefore, the development of organic photovoltaics materials with high *ε*_r_ would be expected to break through the limitations of the PCEs for current OPVs.^13–19^

In recent year, many researchers have developed high *ε*_r_ organic photoluminescence material by introducing fluorine atoms,^16,20–22^ (oligo)ethylene glycol^17,23–27^ or ω-CN substituted alkyl side chain,^14,28–33^ For example, Maes *et al.* reported a series of donner–accepter (D–A) type conjugated polymers (CPs), the alkyl side chains were replaced with glycol side chains, while the *ε*_r_ was increased from 3.0 to 6.3. As a result, the PCEs of the BHJ-OPVs from the polymers increased from 3.30 to 4.42.^33^ Jen *et al.* verified that the replacement of the alkyl side chains with the ω-CN substituted alkyl in conjugated polymers, would increase the *ε*_r_, thus denote to the suppression bimolecular recombination in the BHJ-OPVs from the polymers.^14^ In 2014, Hou *et al.*, introduced fluorine atoms into the side chain of BDT, synthesised four polymers and named PVT-0f, PVT-1F, PVT-2F and PVT-F. It was found that an increase in the number of fluorine atoms leads to a decrease in the HOMO energy level and an increase in the *V*_OC_ from 0.56 V to 0.78 V.^18^ Recently, Ding and Gao *et al.*^34^ demonstrated that the introduction of fluorine atoms into a polymer increased its *ε*_r_ from 3.15 to 4.22, resulting in a power conversion efficiency of 17.01%.^35^ However, researchers have rarely considered the effect of continuous insertion of fluorine atoms on *ε*_r_ of the conjugated polymers.

Conjugated polymers based on gelled aromatic ring family elements are well delocalized π–π conjugated along the main chain. Compared with benzodithiophene (BDT), dithieno[2,3-*d*;2′,3′-*d*′]benzo[1,2-*b*;4,5-*b*′]dithiophene (DTBDT) has a larger conjugate length and surface area, which can reduce the self-assembly of the polymer system and improve carrier mobility.^36^ In 2013, Hou *et al.* introduced alkylthiophene into the side chain of DTBDT, and compared it with BDT in terms of the size and length of conjugated surface.^37^ The larger conjugate surface area and longer conjugate length can effectively improve the charge mobility and reduce the polymer band gap. It is concluded that DTBDT derivatives have good development prospects in the construction of D–A type narrow band gap copolymers. For example, in 2021 Wei *et al.* synthesized a new series of polymer donors consisting of DTBDT and 2,2′-bithiophene-3,3′-dicarboximide (BTI) units, the optimized OSCs based on PDTBDT-T-Cl:Y6 achieved a PCEs of 15.63%.^38^ And in 2022, Zhou *et al.* synthesized conjugated polymers which copolymerized DTBDT with the electron-deficient unit of dithieno[3′,2′:3,4;2′′,3′′:5,6]benzo[1,2-*c*][1,2,5]thiadiazole (DTBT), the PCEs based on PE56-CB:Y6 achieved impressive 16.11% which is the highest value among DTBDT-based polymers.^39^ However, the PCEs of OPVs based on DTBDT derivatives is still relatively poor compared with that of the benzo[1,2-*b*;4,5-*b*′]dithiophenes (BDT)-based analog. Thus, the development of new structure is indispensable.

In this work, we synthesis three alternating conjugated polymers named PDTBDT-0F-BTs, PDTBDT-2F-BTs and PDTBDT-6F-FBTs. By introducing different numbers of fluorine atoms in each repeat unit, we investigate the influence on the optical, aggregation, *ε*_r_, and photovoltaic properties of the polymers. The results showed that the introduction of fluorine atoms into the conjugated polymers leads to a decrease in the HOMO energy levels and an increase in *V*_OC_, *ε*_r_ and charge mobilities. As a result, the PCEs of the OPVs based on polymer:PC_71_BM was proved from 1.69 for PDTBDT-0F-BTs to 1.89 for PDTBDT-2F-BTs and then to 5.28 for PDTBDT-6F-FBTs. Thus, we come to a conclusion that the strategy of introduced fluorine atoms can effectively increase *ε*_r_ of the polymer donors and further improve the PCEs of the OPVs from them.

## 2. Materials and methods

### 2.1. Materials

All reagents, unless otherwise specified, were purchased from Energy Chemical, Aladdin, (Shanghai, China), Sigma-Aldrich Co. and Tokyo Chemical Industry Co., and used as received. 2,7-Bis(trimethylstannyl)-5,10-di(4,5-didecylthieno-2-yl)-dithieno[2,3-*d*;2′,3′-*d*′]benzo[1,2-*b*;4,5-*b*′]dithiophene (DTBDT-Sn) was synthesized by the procedure reported in the reference,^40,41^ the three conjugated polymers were synthesized by the procedure reported in the ref. 40 and presented in the ESI.† And their molecular weights, absorption, electrochemical and aggregation characteristics were characterized^40,42,43^ and presented in the ESI.†

### 2.2. Synthesis of the copolymers

The synthesis of PDTBDT-0F-BTS is identical to that of ESI.† A black solid of 158.87 mg was obtained with a yield of 74%. *M*_n_ = 42.8 kDa, PDI = 2.51. The synthesis of PDTBDT-2F-BTS is identical to that of ESI.† A black solid of 155.03 mg was obtained with a yield of 71%. *M*_n_ = 45.5 kDa, PDI = 2.58. The synthesis of PDTBDT-6F-FBTS is identical to that of ESI.† A black solid of 169.35 mg was obtained with a yield of 76%. *M*_n_ = 32.5 kDa, PDI = 2.11.

## 3. Results

### 3.1. Synthesis and characterization of the monomers and copolymers

**Scheme 1:**
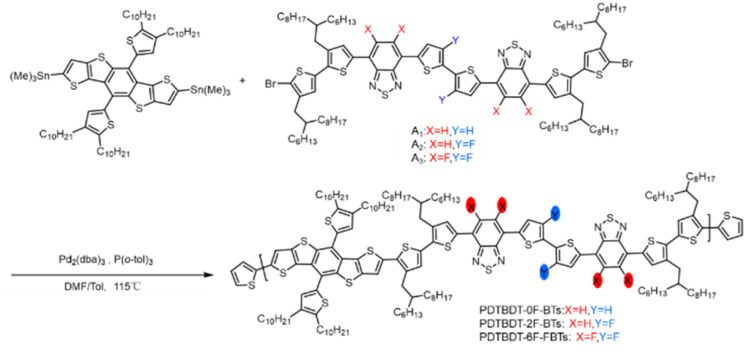
Synthesis routes of the copolymers.

The conjugated copolymers were synthesized according to Scheme 1. A was prepared according to the procedure reported in the literature.^44^ The synthesis involved a Stille coupling reaction of DTBDT-Sn and (3,4′-bis(2-hexyl-decyl)-2,2′-dithiophene-5-yl)-substituted 2,1,3-benzothiadiazole or 5,6-difluoro-2,1,3-benzothiadiazole derivatives with 2,2′-dithiophene or 3,3′-difluoro-2,2′-dithiophene as π-linkers, the detailed synthesis of the monomers being presented in the ESI.† The obtained compound was characterized by ^1^H NMR (Fig. S1–S4, ESI†). The synthetic route of the 2,1,3-benzothiadiazole derivatives is outlined in Scheme S1 of ESI.† The DTBDT-Sn and A_1_ (A_2_ or A_3_) as two alternating units, the copolymers of PDTBDT-0F-BTs, PDTBDT-2F-BTs and PDTBDT-6F-FBTs were prepared and processed according to our reported process in Scheme 1. The *M*_n_ were 42.8 kDa, 45.5 kDa and 32.5 kDa and PDI were 2.51, 2.58 and 2.11 of the copolymers, respectively, and were summarized in Table 1. The thermal decomposition temperature (*T*_d_, 5% weight loss) of the polymers were about 398 °C under N_2_ flow, respectively (Fig. S5, ESI†). This indicates that the copolymer exhibited good thermal stability.

**Table 1:** Optoelectronic parameters of the copolymers

Copolymers	*λ* ^sol^_max_ (nm)	*λ* ^film^_max_ (nm)	*λ* ^film^_onset_ (nm)	*E* ^opt^_g_ a (eV)	*φ* ^onset^_ox_ (eV)	HOMO^b^ (eV)	LUMO^c^ (eV)
PDTBDT-0F-BTs	574	600	734	1.69	0.62	−5.32	−3.60
PDTBDT-2F-BTs	575	594	743	1.67	0.71	−5.41	−3.74
PDTBDT-6F-FBTs	590	606	747	1.66	0.77	−5.50	−3.82

a Optical band gap were estimated by the empirical formula of *E*^opt^_g_ = 1240/*λ*^film^_onset_.

b *E*_HOMO_ were calculated from the formula of *E*_HOMO_ = −(*φ*^onset^_ox_ + 4.70) (eV).

c *E*_LOMO_ were estimated from the formula of *E*_LUMO_ = *E*_HOMO_ + *E*_g_ (eV).

### 3.2. Absorption properties and aggregation characteristics of the copolymers

**Fig. 1:**
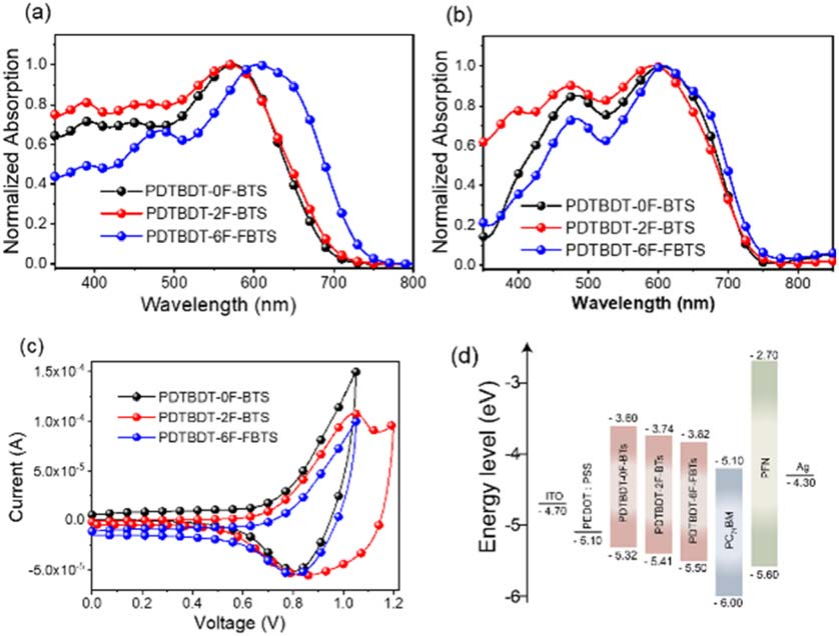
Absorption spectra of the copolymer in solution (a) and in solid film (b). Cyclic voltammogram (c) and energy level schematic (d) of material in this work.

The copolymers were found to be soluble in the main solvents used for the processing of OPV devices. Fig. 1 showed the normalized absorption spectra of the copolymers in solution and solid film state. The absorption data are summarized in Table 1. All the polymers showed a board and strong absorption in the range of 300–750 nm in CB solution (Fig. 1a). The PDTBDT-0F-BTs, PDTBDT-2F-BTs and PDTBDT-6F-FBTs showed the maximal absorption peaks at around 574 nm, 575 nm and 590 nm, respectively, which were mainly caused by the intermolecular charge transfer (ICT) transition, and the secondary peaks at around 390 nm, 388 nm and 492 nm, were ascribed to the π–π* transition from the main chain units of the polymer backbone, respectively. Compared to the solution, the maximal absorption peaks of the PDTBDT-0F-BTs, PDTBDT-2F-BTs and PDTBDT-6F-FBTs thin films have different degree of red-shifts of 36 nm, 19 nm and 16 nm, respectively (Fig. 1b). For Fig. 1a and b, the 0–0 and 0–1 peaks can be observed, where the 0–0 peak indicates the aggregation state of the molecules, and the higher the peaks indicate the stronger aggregation. The shoulder peaks of PDTBDT-6F-FBTs are slightly higher than those of PDTBDT-2F-BTs and PDTBDT-0F-BTs, and the intensity of the 0–0 peaks/intensity of the 0–1 peaks decreases from PDTBDT-2F-BTs to PDTBDT-6F-FBTs, which indicates that the aggregation of PDTBDT-6F-FBTs is weaker in solution, and that the polymers in the thin film state show better aggregation properties compared to the solution state.

The optical band gap (*E*^opt^_g_) of PDTBDT-6F-FBTs film is calculated to be 1.66 eV, according to the formula *E*^opt^_g_ = 1240/*λ*^film^_onset_, which is narrower relative to PDTBDT-2F-BTs (1.67 eV) and PDTBDT-0F-BTs (1.69 eV). Fluorination of copolymer red-shifts the film absorption and reduces the band gap. To illustrate the aggregation behavior of the polymers in solution more clearly, we also inspected the temperature-dependent absorption (TD-Abs) spectra in chlorobenzene (CB) solution (Fig. S6, ESI†). When the temperature increased the absorption peak of ICT was blue shifted. It was showed that absorbance decreases was 4.5% for PDTBDT-0F-BTs (24 nm), 3.2% for PDTBDT-2F-BTs (26 nm) and 12.4% for PDTBDT-6F-FBTs (51 nm), respectively. The blue-shifted of the ICT absorption peak and the decrease in absorbance strength of PDTBDT-6F-FBTs were more obvious, indicated stronger aggregation compared with the PDTBDT-0F-BTs and PDTBDT-2F-BTs in the solution.

### 3.3. Electrochemical properties, dielectric constant and GIWAXS of the copolymers

The effect of the introduction of fluorine atoms on the energy levels of the copolymers was investigated using cyclic voltammetry (CV). The PDTBDT-0F-BTs, PDTBDT-2F-BTs and PDTBDT-6F-FBTs provided the oxidation potentials (*φ*^onset^_ox_) of 0.62 V, 0.71 V and 0.77 V (Fig. 1c), respectively. And the HOMO and lowest unoccupied orbital (LUMO) energy levels of the PDTBDT-0F-BTs, PDTBDT-2F-BTs and PDTBDT-6F-FBTs were determined by the empirical formulae of *E*_HOMO_ = −(*φ*^onset^_ox_ + 4.70) (eV) and *E*_LUMO_ = *E*_HOMO_ + *E*^opt^_g_ (eV), were about −5.32/−3.60 eV, −5.41/−3.74 eV and −5.50/−3.82 eV, respectively. For more intuitive comparisons, the energy level diagram of the copolymers and PC_71_BM has been described in Fig. 1d. In parallel, the density functional theory (DFT) calculations were carried out at the B3LYP/6-31G* basis set by using the Gaussian 09 program suite. The calculated HOMO of the PDTBDT-0F-BTs, PDTBDT-2F-BTs and PDTBDT-6F-FBTs were at −4.64 eV, −4.65 eV and −5.11 eV and the LUMO at −2.88 eV, −2.94 eV and −2.98 eV, (Fig. 3) respectively, which were consistent with the results obtained from the CV measurements. It was noted that as the number of fluorine atoms in the conjugated polymer increases, the HOMO of the polymer decreases.

**Fig. 2:**
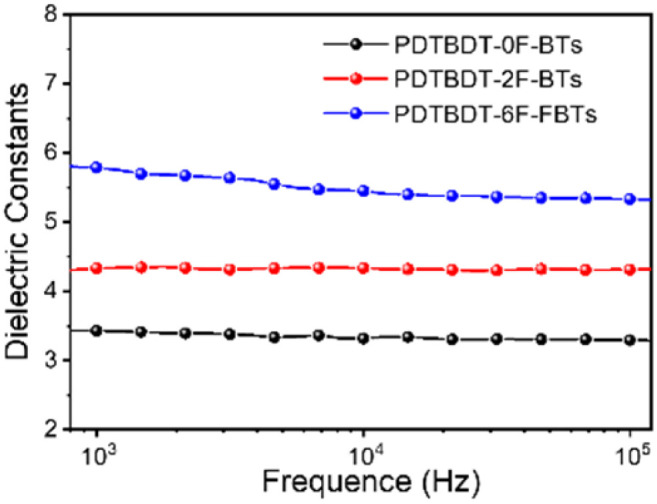
Variation of the relative dielectric constants of the copolymers with frequency.

**Fig. 3:**
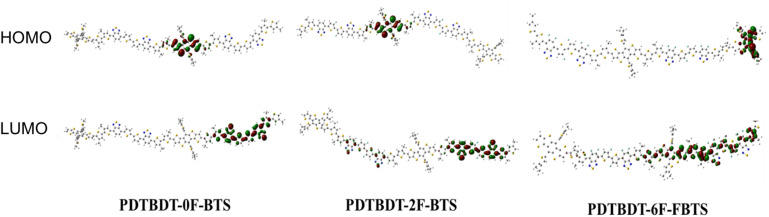
DFT-calculated frontier molecular orbitals of PDTBDT-0F-BTS, PDTBDT-2F-BTS and PDTBDT-6F-FBTS.

To investigate the effect of the number of fluorine atoms on *ε*_r_,^45^ devices based on three copolymers were prepared and *ε*_r_ was calculated using eqn S1.†Fig. 2 showed that the *ε*_r_ are 3.4 for PBDT-2F-BTs, 4.3 for PBDT-0F-BTs and 5.8 for PBDT-6F-FBTs at 1 kHz. The results showed that the continuous addition of fluorine atoms to the repeating units of the copolymer leads to an increase in the *ε*_r_ of the copolymer.

**Fig. 4:**
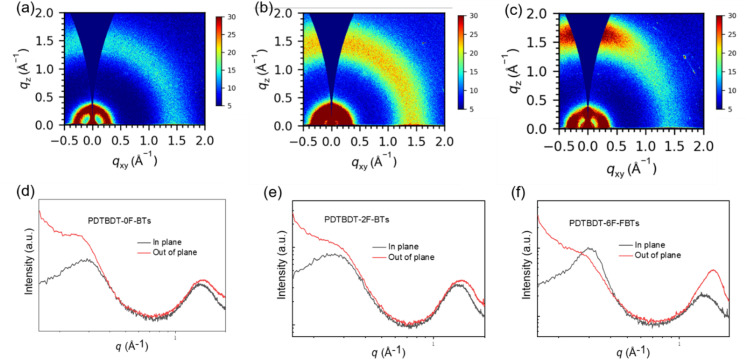
2D GIWAXS patterns (a)–(c) and corresponding in-plane and out of-plane line cuts (d)–(f) for PDTBDT-0F-FBTs (a) and (d), PDTBDT-2F-FBTs (b) and (e) and PDTBDT-6F-FBTs (c) and (f) blend films.

Grazing-incidence wide-angle X-ray scattering (GIWAXS) refers to a testing technique based on the interaction of X-rays with matter, which allows further probing of crystallinity and orientation.^57,58^ Need to study out-of-plane (out-of-plane, perpendicular to the substrate) and in-plane (in-plane, parallel to the substrate) stacking. From the derived 1D data, the *d*-spacing (*d* = 2π/*q*), half-peak widths, and coherence lengths of the major peaks can be calculated. The 2D scattering images and the associated line-cut profiles of three polymer film showed in Fig. 4. Table S1† summarized associated data. As can be seen from the Fig. 4, in the films based on PDTBDT-0F-BTs, PDTBDT-2F-BTs and PDTBDT-6F-FBTs edge orientation and face orientation coexist, with 1.47 Å^−1^, 1.46 Å^−1^ and 1.56 Å^−1^ for the 010 peaks, 0.28 Å^−1^, 0.26 Å^−1^ and 0.30 Å^−1^ for the 100 peaks, the π–π stacking distances (*d*_π_) are calculated to be 4.27 Å, 4.30 Å and 4.03 Å, the lamellar stacking distances (*d*_L_) are 22.44 Å, 24.17 Å and 21.08 Å, the crystal coherence lengths (CCL) are 27.43 Å and 30.75 Å and 51.71 Å. The FF value of the films based on PDTBDT-6F-FBTs was increased by 19.4% due to tighter π–π stacking, superior crystallinity and ideal molecular orientation and hence improved charge mobility.

### 3.4. Hole mobilities of the blend films from the copolymers

**Fig. 5:**
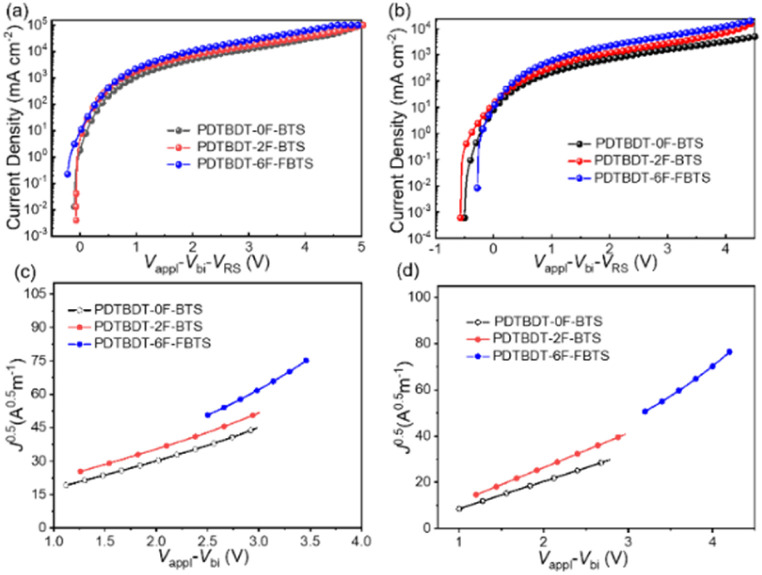
*J*–*V* (a) and *J*^0.5^–*V* (c) curves for hole-transport devices, *J*–*V* (b) and *J*^0.5^–*V* (d) curves for electron-transport devices.

In order to evaluate the electron transport capability, we investigated the hole mobility of three polymer films in devices with ITO/PEDOT:PSS/polymer:PC_71_BM/MoO_3_/Ag, used the space charge limiting current (SCLC) method.^46–48^ The charge transport characteristics of the copolymers paired with PC_71_BM blend films with a weight ratio of 1 : 2, and the *J*–*V* behavior the electron-only devices with configuration of ITO/ZnO/blend films/PFN/Al and hole-only devices with the configuration of ITO/PEDOT: PSS/blend films/MoO_3_/Ag, respectively, are given in Fig. 5, ESI,† respectively. The hole mobilities (*μ*_h_) of the blend films of PDTBDT-0F-BTs/PC_71_BM, PDTBDT-2F-BTs/PC_71_BM and PDTBDT-6F-FBTs/PC_71_BM were approximately 1.06 × 10^−4^, 1.09 × 10^−4^ and 1.55 × 10^−4^ cm^2^ V^−1^ s^−1^ (Fig. 5c), and the electron mobilities (*μ*_e_) were about 9.97 × 10^−5^ and 1.08 × 10^−4^, 1.65 × 10^−4^ cm^2^ V^−1^ s^−1^ (Fig. 5d), respectively (Table 2). It was found that the hole and electron mobility of their blend films decreased sequentially with the increase in the number of fluorine atoms.

**Table 2:** Parameters of the photovoltaic cells from the polymers paired with PC_71_BM

Active layer (w : w, 1 : 2)	*V*_OC_ (V)	*J*_SC_ (mA cm^−2^)	FF (%)	PCE (%)	*ε*_r_	*μ* (cm^2^ V^−1^ s^−1^)	
						*μ*_e_	*μ*_h_
PDTBDT-0F-BTs:PC_71_BM	0.82	4.58	42.6	1.69	3.4	9.97 × 10^−5^	1.06 × 10^−4^
PDTBDT-2F-BTs:PC_71_BM	0.83	4.74	47.0	1.89	4.3	1.08 × 10^−4^	1.09 × 10^−4^
PDTBDT-6F-FBTs:PC_71_BM	0.86	8.76	66.4	5.28	5.8	1.65 × 10^−4^	1.55 × 10^−4^

### 3.5. Photovoltaic characteristics and optical modeling of the device from the copolymers

**Fig. 6:**
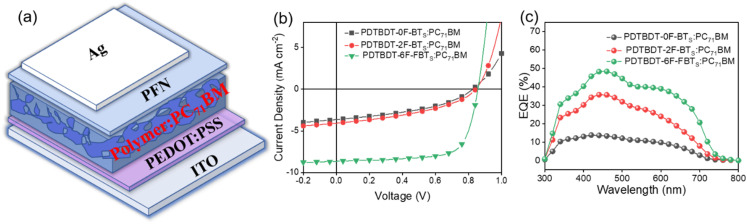
Device structure diagram (a); the optimized current density–voltage (*J*–*V*) curves (b); the EQE spectra (c).

To compare the potential application of the three copolymers as donor materials in photovoltaic properties, we used PC_71_BM as the acceptor to fabricate OPVs with the following conventional structure: ITO/PEDOT:PSS/polymer:PC_71_BM/PFN/Ag.^49^ (Fig. 6a) The current density–voltage (*J*–*V*) curves are shown in Fig. 6b and the photovoltaic parameters are summarized in Table 2. The device is optimized by adjusting the donor/acceptor (D/A) weight ratios and by adding solvent additives. The active layers of polymer:PC_71_BM was selected with a D/A ratio of 1 : 2 in a total concentration of 21 mg mL^−1^ in chlorobenzene (CB). By adding 2% DIO (DIO/CB, v/v) as the solvent additive to further optimize the PCE, the PDTBDT-6F-FBTs show the best PCE of 5.28% compared to PDTBDT-0F-BTs (1.69%), PDTBDT-2F-BTs (1.89%), with open-circuit voltages (*V*_OC_) of 0.82 V, 0.83 V and 0.86 V, short-circuit current densities (*J*_SC_) of 8.76 mA cm^−2^, 4.58 mA cm^−2^ and 4.74 mA cm^−2^, and fill factors (FF) of 66.4%, 42.6% and 47.0% (Table 2). As the number of fluorine atoms increases, the performance of the device is improved and its *V*_OC_, PCE, *J*_SC_ and FF gradually increase. The enhancement of the *J*_SC_ and FF of the devices from the copolymers could result from the gradual increase of the *ε*_r_ of the copolymers by successive insertion of the fluorine atoms into the copolymers. The external quantum efficiency (EQE) curves of the devices were characterized according to the procedure reported in reference,^50,51^ and the photo-response profiles of the optimal devices in the ranges of 300–750 nm for the copolymers, were observed. The higher *J*_SC_ in the PDTBDT-6F-FBTs-based device was due to its broadened absorption and higher EQE value (Fig. 6c).

**Fig. 7:**
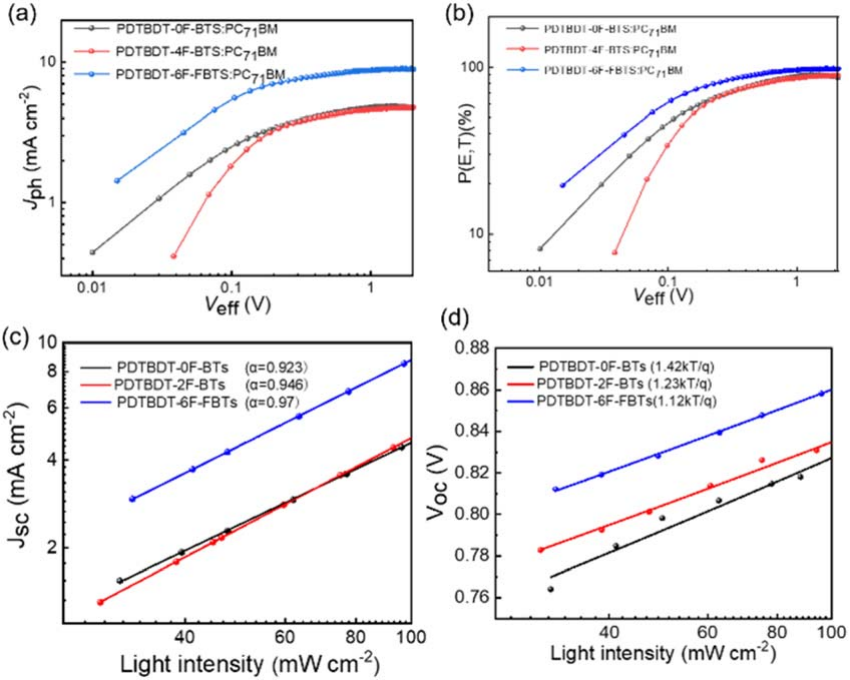
The curves of *J*_ph_–*V*_eff_ (a); *P*(*E*, *T*)–*V*_eff_ plots (b); *V*_oc_ (c) and *J*_sc_ (d) *versus* the natural logarithm of light intensities of optimised devices characteristics of the inverted PVCs from the three copolymers with 2% DIO additive under AM 1.5G illumination (100 mW cm^−2^).

To investigate the exciton dissociation and charge collection properties, Fig. 7a reveals the *J*_ph_–*V*_eff_ curves for these devices. Where *J*_ph_ = *J*_L_ − *J*_D_, *J*_D_ and *J*_L_ were obtained by testing the device in sunlight and darkness. And *V*_eff_ = *V*_0_ − *V*_appl_, where *V*_0_ and *V*_appl_ represent the voltage at *J*_ph_ = 0 and the voltage imposed on the device, respectively.^52^ As shown in Fig. 7a, the *J*_ph_ of the devices from PDTBDT-0F-BTs, PDTBDT-2F-BTs and PDTBDT-6F-FBTs was in the process of linear growth when *V*_eff_ < 0.2 V, and then saturated when the *V*_eff_ > 1, suggesting that most of the excitons had been dissociated with the help of a large reverse bias. Moreover, the saturation rate of *J*_ph_ of PDTBDT-6F-FBTs was faster compared with PDTBDT-0F-BTs and PDTBDT-2F-BTs. And the charge dissociation probability *P*(*E*, *T*) of the devices were about 86.12%, 88.87% and 98.12% respectively, shown in Fig. 7b. In addition, to study the charge recombination behavior of devices based on PDTBDT-0F-BTs:PC_71_BM, PDTBDT-2F-BTs:PC_71_BM, PDTBDT-6F-FBTs:PC_71_BM with the active layer. We studied the dependence of *J*_SC_ and *V*_OC_ on light intensity (*P*), as shown in Fig. 7c and d. In general, the parameters *J*_SC_ and *P* follow the relationship between *J*_SC_ ∝ *P*^*α*^, with *α* closer to 1 indicating a great inhibition of the bimolecular recombination.^53,54^ The *α* values of the devices from PDTBDT-0F-BTs, PDTBDT-2F-BTs and PDTBDT-6F-FBTs were 0.923, 0.946 and 0.970, respectively. There is a competition between bimolecular recombination and trap-assisted recombination in terms of *P*(light) effect on *V*_OC_. The results show high FF and *J*_SC_ values based on the PDTBDT-6F-FBTs device due to its efficient carrier transport and its negligible bimolecular recombination.

*V*_OC_ and *P*(light) satisfy the relationship 
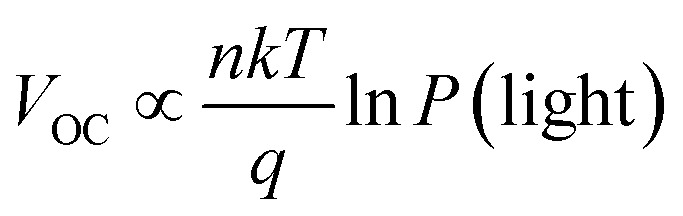
 where *n* is the slope, when *n* is close to 1, it indicates that bimolecular recombination is dominant, and when *n* is close to 2 it indicates a trap-assisted recombination.^55^ For PDTBDT-0F-BTs, PDTBDT-2F-BTs and PDTBDT-6F-FBTs the *n* values of the devices are 1.42 *kT*/*q*, 1.23 *kT*/*q* and 1.12 *kT*/*q*, respectively, indicating the possible presence of molecular recombination and trap-assisted recombination in these devices. Trap-assisted recombination is inhibited in these devices. The *n* value of PDTBDT-6F-FBTs as the active layer further decreases and approaches 1, which suggests a decrease in bimolecular recombination and a further inhibition of the rap-assisted recombination process.

### 3.6. Impedance spectroscopy

**Fig. 8:**
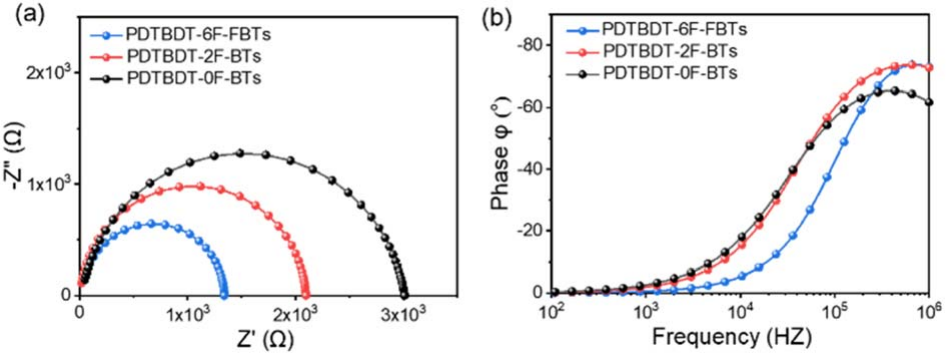
Impedance spectra measured in the dark at different bias as indicated. (a) Nyquist diagram; (b) Bode phase diagram.

To further investigate the charge transport capacity, we measured the impedance spectrum under dark conditions. Fig. 8 showed the Nyquist diagrams and the Bode phase diagrams of the interfacial charge transfer time constant (*τ*) of the three copolymers. The resulting Nyquist spectra were fitted using the equivalent circuit included as inset in Fig. 8a. In this circuit, RS accounts for the series resistance of the system, the transport of electrons is represented by means of *R*_1_, *C* is the dielectric capacitor of the device and CPE is a constant phase element acting as chemical capacitor produced by photo-generated charge accumulation. Fig. 8a showed Nyquist diagrams of devices with PDTBDT-0F-BTS:PC_71_BM, PDTBDT-2F-BTS:PC_71_BM and PDTBDT-6F-FBTS:PC_71_BM respectively. *R*_1_ of PDTBDT-0F-BTS:PC_71_BM, PDTBDT-2F-BTS:PC_71_BM and PDTBDT-6F-FBTS:PC_71_BM devices were 3005 Ω, 2150 Ω and 1335 Ω respectively. The results, where *R*_1_ of PDTBDT-6F-FBTS:PC_71_BM was significantly reduced, exhibited that the conductivity had been enhanced while the number of the fluorine atoms in each repeat units had been increased, which facilitated charge transfer.^50,56^Fig. 8b showed the peak characteristic frequency (*f*_max_) and the Bode phase diagram of the *τ*, according to formula (1)

1 *f*_max_ ∝ 1/*τ*

The characteristic *f*_max_ of the devices with PDTBDT-0F-BTS:PC_71_BM, PDTBDT-2F-BTS:PC_71_BM and PDTBDT-6F-FBTS:PC_71_BM are 354 200 Hz, 559 645 Hz, and 688 752 Hz respectively. The result shows that the photogenerated carriers of the PDTBDT-6F-FBTS device can reach the cathode in a shorter time, thus improving the efficiency of electron collection.

### 3.7. Morphological properties

**Fig. 9:**
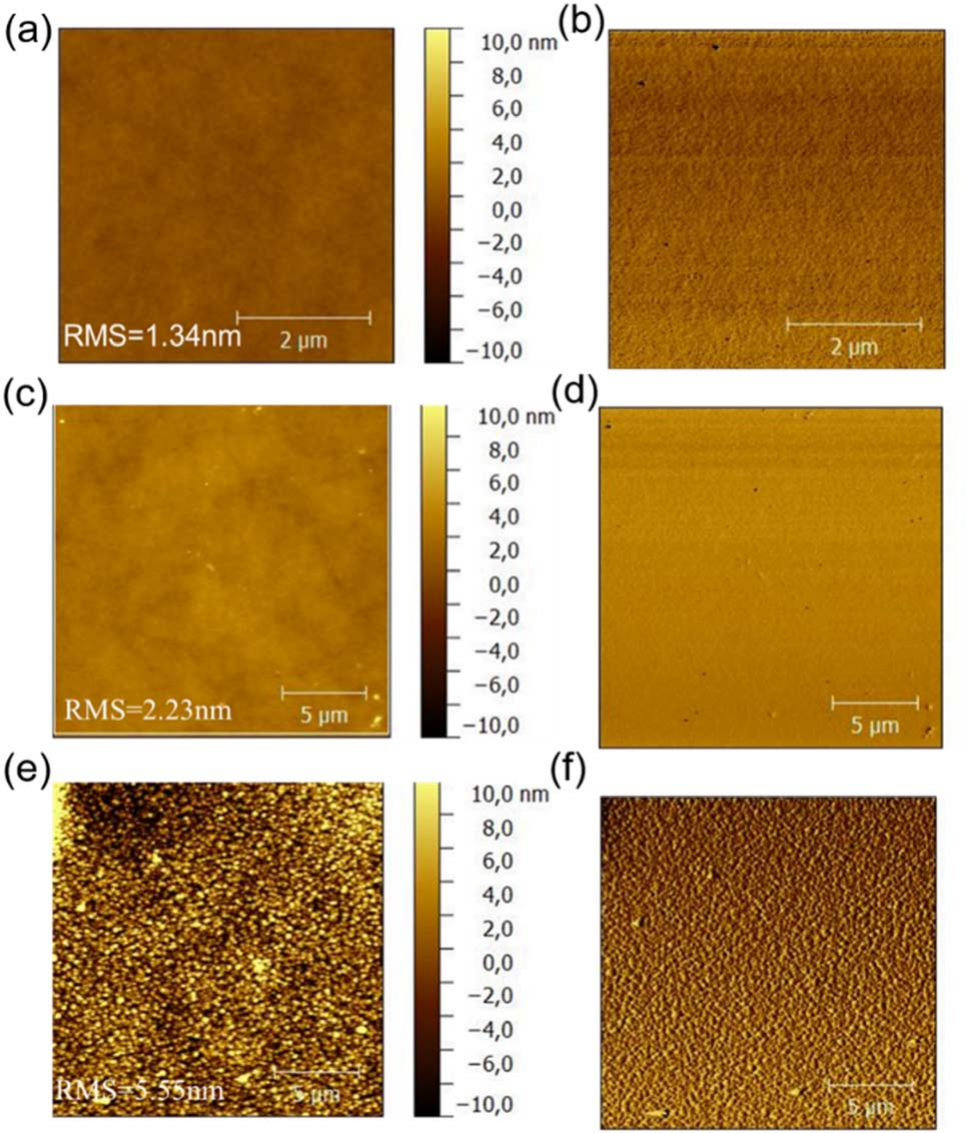
AFM height images and phase images of the PDTBDT-0F-BTS:PC_71_BM (a) and (b), PDTBDT-2F-BTS:PC_71_BM (c) and (d) and PDTBDT-6F-FBTS:PC_71_BM (e) and (f).

The morphology of the active layer which will directly affect the photovoltaic performance of organic solar cells was characterized with the atomic force microscopy (AFM), the resulted height images of three blend films were shown in Fig. 9. PDTBDT-0F-BTS:PC_71_BM, PDTBDT-2F-BTS:PC_71_BM and PDTBDT-6F-FBTS:PC_71_BM showed relatively uniform morphology with corresponding root mean-square (RMS) roughness values of 1.34, 2.23 and 5.55 nm, respectively. We noticed that the surface roughness and domain size of the copolymers gradually increased as the number of fluorine atoms in each repeating unit increased from 0 to 2 and then up to 6. This was attributed to the decrease in solubility of the polymer due to the increase in the number of fluorine atoms. Meanwhile, PDTBDT-6F-FBTS:PC_71_BM showed the best suitable phase separation, favouring charge transport and collection as well as the reduced bimolecular recombination, the *J*_SC_ and FF were improved with the increase in the number of fluorine atoms of the repeating units in the copolymer and PC_71_BM blend films.

## 4. Conclusion

In summary, three alternating copolymers named PDTBDT-0F-BTs, PDTBDT-2F-BTs, and PDTBDT-6F-FBTs, containing the varied numbers of fluorine atoms (from 0 to 2 and then up to 6) in each repeat units were synthesized. On the one hand, the fluorination polymers can effectively deepen the HOMO levels from −5.32 eV to −5.41 eV, and then down to −5.50 eV, while the numbers of the fluorine atoms in each repeat units were increased. An increase in the number of fluorine atoms leads to an increase in the value of the *ε*_r._ On the other hand, impedance spectra and morphological features are studied in detail, the crystalline properties was best based on PDTBDT-6F-FBTs. These results show that continuous insertion of fluorine atoms in the copolymer leads to increases in charge mobility, dielectric constant, HOMO energy levels and better crystalline properties, thus denote to improvement of the performance of the BHJ-OPVs from the polymers, which has PCE of 5.28%.

## Author contributions

Pengzhi Guo: conceptualization, formal analysis, writing-original draft; Xuemei Gan: methodology, visualization; Sheng Guan: investigation, resources; Peili Gao: writing-review & editing; Qian Wang: validation; Furong Shi: software; Yuan Zhou: supervision; Chenglong Wang: data curation; Yangjun Xia: funding acquisition, project administration.

## Conflicts of interest

The authors declare that they have no known competing financial interests or personal relationships that could have appeared to influence the work reported in this paper.

## Supplementary Material

RA-014-D4RA01104J-s001
