# Supplementary material for: Effect of fluorine on the photovoltaic properties of 2,1,3-benzothiadiazole-based alternating conjugated polymers by changing the position and number of fluorine atoms
Source: RSC Adv. 2024 Apr 11;14(17):11659–67. doi: 10.1039/d4ra01104j (PMC11007488; doi:10.1039/d4ra01104j)
Supplement: RA-014-D4RA01104J-s001 [file RA-014-D4RA01104J-s001.pdf]

Electronic Supplementary Material (ESI) for RSC Advances.
This journal is © The Royal Society of Chemistry 2024

Supporting Information

Effect of fluorine on the photovoltaic properties of 2,1,3-benzothiadiazole-based alternating conjugated polymers by changing the position and number of fluorine atoms

Pengzhi Guo,*[a] Xuemei Gan,[a] Sheng Guan,[a] Peili Gao,[b] Qian Wang,[b] Furong Shi,[b] Yuan Zhou,[a] Chenglong Wang,[a] and Yangjun Xia,[b]

[a.] National Green Coating Technology and Equipment Engineering Technology Research Center, Lanzhou Jiaotong University, Lanzhou 730070, China;

[b.] Organic Semiconductor Materials and Applied Technology Research Center of Gansu province, School of Material Science and Engineering, Lanzhou Jiaotong University, Lanzhou 730070, China

* Corresponding author.

E-mail addresses: shxygpz@126.com (P.G.);

Characterization

^{1}H NMR spectra were recorded on a BRUKER 500 MHz AVANCE NEO spectrometer operating at 500 MHz. Analytical gel permeation chromatography (GPC) was performed on a Waters GPC 2410 in tetrahydrofuran (THF) via a calibration curve of polystyrene standard. UV-visible absorption spectra were measured on a UV-2550 spectrophotometer (Shimadzu). Thermogravimetric analysis (TGA) was measured on a TGA 2050 (TA instruments) thermal analysis system under a heating rate of 10 $^{o}C\cdot min^{-1}$ and a nitrogen flow rate of 20 $mL\cdot min^{-1}$. The cyclic voltammetry (CV) characteristics of the polymers were measured on a CHI 600D electrochemical workstation (Shanghai Chenhua Co.) at a scan rate of 50 mV/s in a nitrogen-saturated mixed solution of acetonitrile containing 0.1 M tetrabutylammonium hexafluorophosphate (Bu_4NPF_6) with glassy carbon electrode, Ag/$AgNO_3$ electrodes and platinum wire as the working, reference and counter electrodes. The redox potential of Fc/Fc^+ was about +0.10 V, and set the absolute energy level of Fc/Fc^+ potential under vacuum to −4.70 eV under above condition. Atomic Force Microscopy (AFM) measurements of the film morphology were imaged by an Agilent 5400 with tapping mode. The capacitance of each film was recorded with an Agilent HP 4278A by applying a small voltage perturbation (20 mV rms) and sweeping frequencies from 1 MHz down to 20 Hz. respectively.

Synthesis of PDTBDT-0F-BTS

Added the purified DTBDT-Sn (108 mg, 0.08 mmol) and A_1 (145.8 mg, 0.08 mmol), 10 mL of toluene and 2 mL of *N,N*-dimethylformide (DMF) were added to a 25 mL two necked bottle. After being flushed with argon for 20 min, the catalyst $Pd_2(dba)_3$ (1.5 mg) and P(*o*-tolyl)$_3$ (3 mg) were added, and the mixture was then purged with argon for 10 min. The solution was stirred and heated to reflux for 48 h under argon atmosphere. At the end of the polymerization, the polymer was end-capped with 2-tributylstannylthiophene and 2-bromothiophene to remove bromo and trimethylstannyl end groups. The reaction was cooled to room temperature, and the mixture was precipitated in methanol and filtered. Further purification was carried out by Soxhlet extraction using the sequence ethanol, acetone, hexane and toluene as the eluents to remove the residual catalyst and oligomers. Following that, the concentrated solutions of the copolymers in toluene were poured into methanol again (300 mL). The

precipitation was collected and dried under vacuum overnight. A black solid of 158.87 mg was obtained with a yield of 74%. Number average molecular weight (M_n) = 42.8 kDa with polydispersity index (PDI) of 2.51.

Synthesis of PDTBDT-2F-BTS

Added the purified DTBDT-Sn (108 mg, 0.08 mmol) and A_2 (145.8 mg, 0.08 mmol), 10 mL of toluene and 2 mL of *N,N*-dimethylformide (DMF) were added to a 25 mL two necked bottle. After being flushed with argon for 20 min, the catalyst $Pd_2(dba)_3$ (1.5 mg) and P(*o*-tolyl)$_3$ (3 mg) were added, and the mixture was then purged with argon for 10 min. The solution was stirred and heated to reflux for 48 h under argon atmosphere. At the end of the polymerization, the polymer was end-capped with 2-tributylstannylthiophene and 2-bromothiophene to remove bromo and trimethylstannyl end groups. The reaction was cooled to room temperature, and the mixture was precipitated in methanol and filtered. Further purification was carried out by Soxhlet extraction using the sequence ethanol, acetone, hexane and toluene as the eluents to remove the residual catalyst and oligomers. Following that, the concentrated solutions of the copolymers in toluene were poured into methanol again (300 mL). The precipitation was collected and dried under vacuum overnight. A black solid of 158.87 mg was obtained with a yield of 71%. Number average molecular weight (M_n) = 45.5 kDa with polydispersity index (PDI) of 2.58.

Synthesis of PDTBDT-6F-BTS

Added the purified DTBDT-Sn (108 mg, 0.08 mmol) and A_3 (145.8 mg, 0.08 mmol), 10 mL of toluene and 2 mL of *N,N*-dimethylformide (DMF) were added to a 25 mL two necked bottle. After being flushed with argon for 20 min, the catalyst $Pd_2(dba)_3$ (1.5 mg) and P(*o*-tolyl)$_3$ (3 mg) were added, and the mixture was then purged with argon for 10 min. The solution was stirred and heated to reflux for 48 h under argon atmosphere. At the end of the polymerization, the polymer was end-capped with 2-tributylstannylthiophene and 2-bromothiophene to remove bromo and trimethylstannyl end groups. The reaction was cooled to room temperature, and the mixture was precipitated in methanol and filtered. Further purification was carried out by Soxhlet extraction using the sequence ethanol, acetone, hexane and toluene as the eluents to remove the residual catalyst and oligomers. Following that, the concentrated solutions of the copolymers in toluene were poured into methanol again (300 mL). The

precipitation was collected and dried under vacuum overnight. A black solid of 158.87 mg was obtained with a yield of 76%. Number average molecular weight (M_n) = 32.85 kDa with polydispersity index (PDI) of 2.11.

Preparation of photovoltaic devices

To evaluate the device photovoltaic performance of PDTBDT-0F-BTs, PDTBDT-2F-BTs and PDTBDT-6F-FBTs, orthotropic device structures with ITO/ PEDOT: PSS/ polymer: $PC_{71}BM$/PFN /Ag. electrode structures were prepared. The ITO glass was first treated by ultrasonic cleaning in deionized water with detergent, deionized water, acetone and isopropyl alcohol in sequence, and then treated in a UV ozone cleaner for 15 min. an aqueous solution of PEDOT: PSS was filtered through a 0.25 μm pore size filter and then spin-coated on pre-cleaned ITO-coated glass at 5000 rpm/s for 30 seconds. Subsequently, the PEDOT: PSS films were annealed in air at 150 °C for 15 min to form films with a thickness of about 30 nm. A mixture of the polymer donor and $PC_{71}BM$ acceptor was dissolved in ultra-dry chloroform and then spin-coated on the PEDOT: PSS layer at 3000 rpm. Subsequently, the organically active layer film was annealed at 110 °C for 10 min in an N_2 environment to form a film with a thickness of about 90 nm. Finally, metallic Ag was physically vapor deposited under a mask at ~10^{-4} Pa in a vacuum

Preparation of SCLC

In order to evaluate the electron transport ability of OSCs based on PDTBDT-0F-BTs, PDTBDT-2F-BTs and PDTBDT-6F-FBTs as active layers, we measured the hole mobility of spin-coated different active layers using the space charge limiting current (SCLC) method with the device structure of ITO/PEDOT: PSS/blend films/MoO_3/Ag, and The ITO glass was first treated with ultrasonic cleaning in detergent-added deionized water, deionized water, acetone and isopropanol in that order, and then treated in a UV-ozone cleaner for 15 min. The aqueous solution of PEDOT: PSS was filtered through a filter with a pore size of 0.25 μm, and then spun-coated on pre-cleaned ITO-coated glass for 30 s at 5000 rpm. The PEDOT: PSS film was then annealed at 155°C in air for 15 minutes to form a film with a thickness of about 30 nm. Subsequently, the PEDOT: PSS films were annealed in air at 155 °C for 15 min to form films with a thickness of about 30 nm. A mixture of polymer donor and $PC_{71}BM$ acceptor (mass ratio 1: 2) was dissolved in ultra-dry chloroform and then spin-coated

on the PEDOT: PSS layer at 3000 rpm. Subsequently, the organically active layer film was annealed at 110 °C for 10 min in an N_2 environment to form a film with a thickness of about 90 nm. MoO_3 was then physically vapor deposited under a mask plate in a vacuum at ~10^{-4} Pa with a thickness of 5 nm. finally, metallic Ag was physically vapor deposited under a mask plate in a vacuum at ~10^{-4} Pa.

Eq. (1)

$$\varepsilon_r = \frac{C}{\varepsilon_0} \times \frac{d}{A} \tag{1}$$

where ε_0 is the permittivity of free space, d is the thickness of the polymer which is approximately 150 nm, A is the area of the device which is 0.1 cm^2.

Eq. (2)

$$\mu = \frac{8L^3 J}{9\varepsilon_0 \varepsilon_r V^2} \tag{2}$$

where J is the current density, ε_0 is the permittivity of free space, ε_r is the relative dielectric constant of the transport medium, V is the internal potential in the devices and L is the thickness of the active layers.

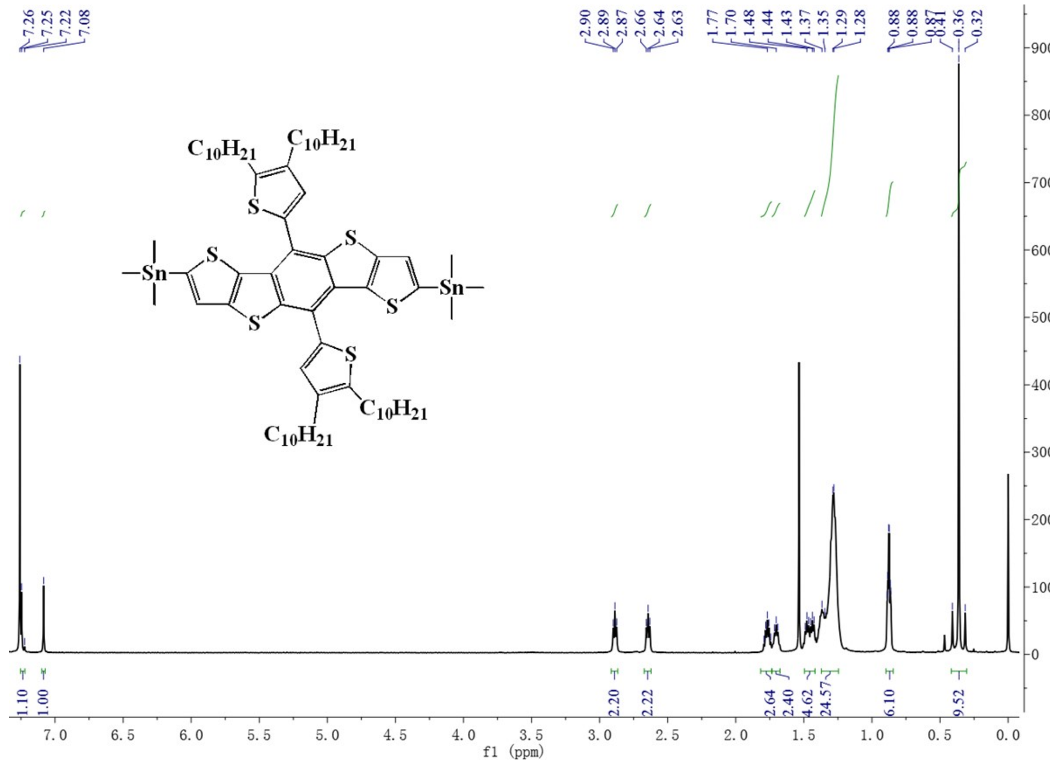

Fig. S1. ^{1}H NMR spectrum of DTBDT-TSn in $CDCl_3$.

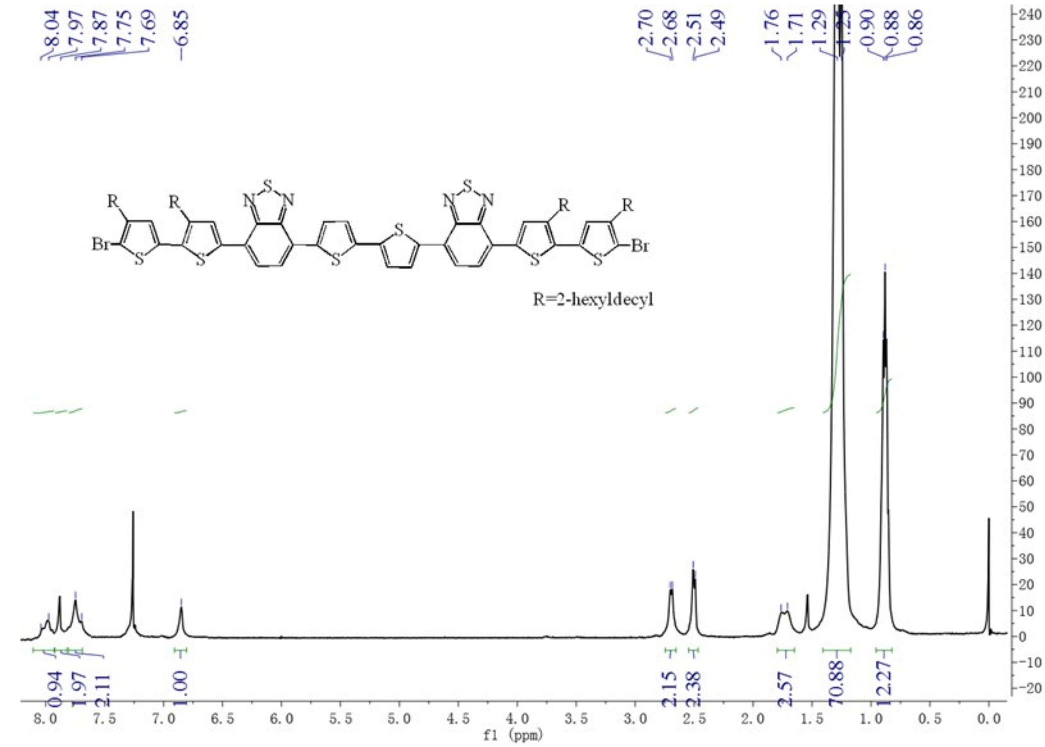

Fig. S2. ^{1}H NMR spectrum of BrDTtBT-TT in $CDCl_3$.

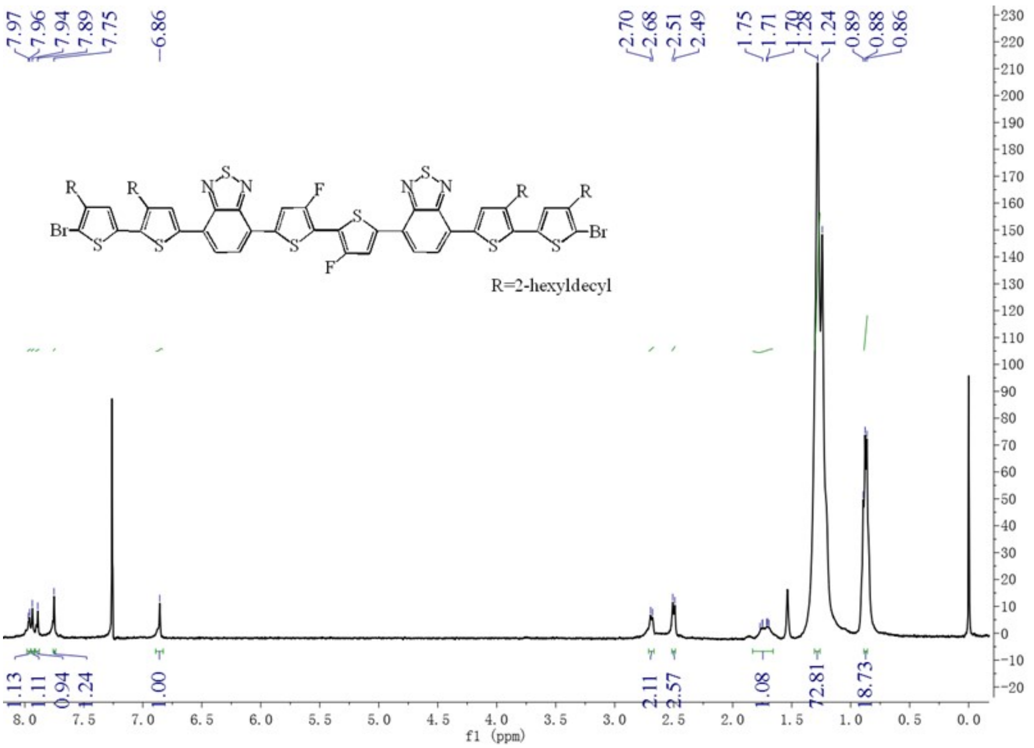

Fig. S3. ^{1}H NMR spectrum of BrDTtBT-FTT in $CDCl_3$.

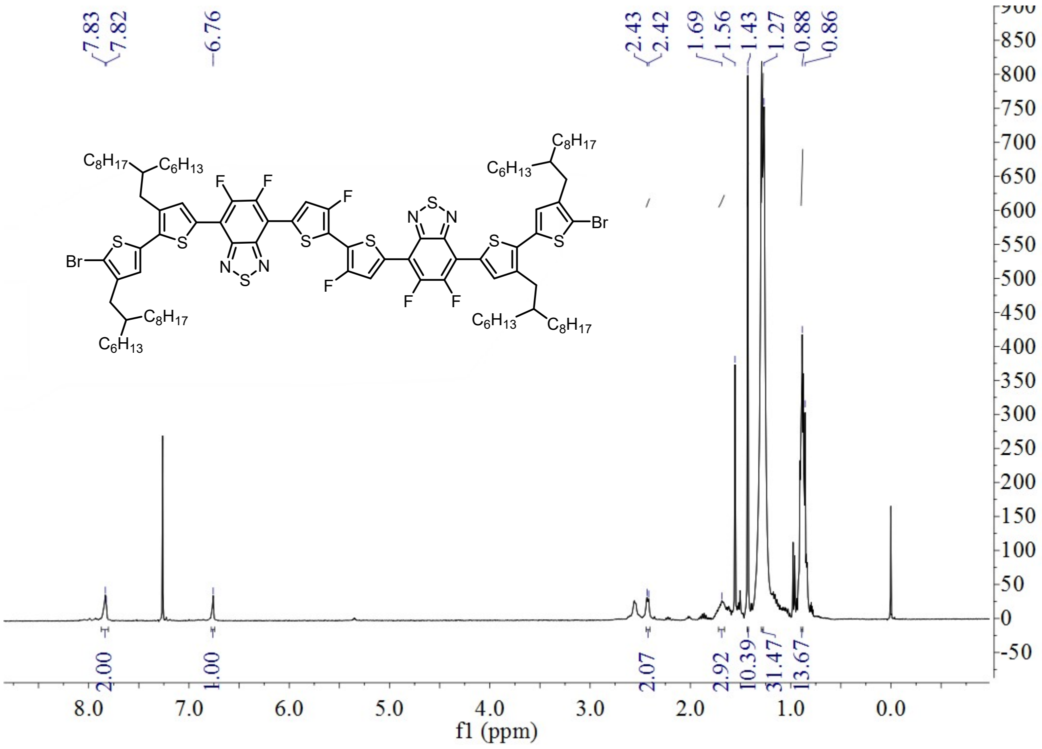

Fig. S4. ^{1}H NMR spectrum of BrDTtFBT-FTT in $CDCl_3$.

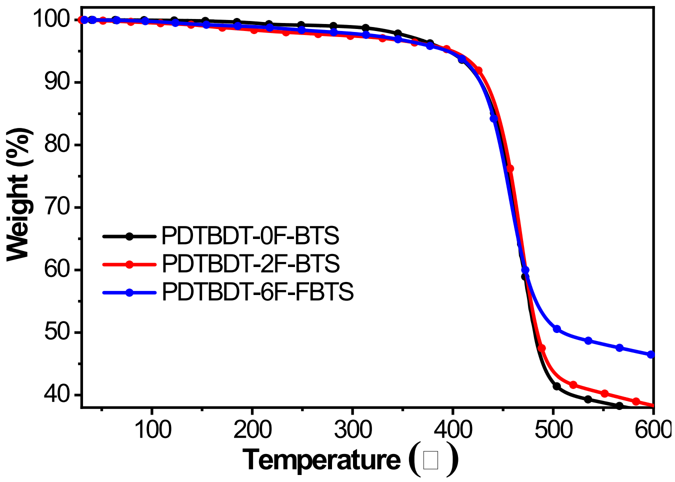

Fig. S5. Thermogravimetric analysis curve of polymers with heating rate of 5°Cmin^{-1}.

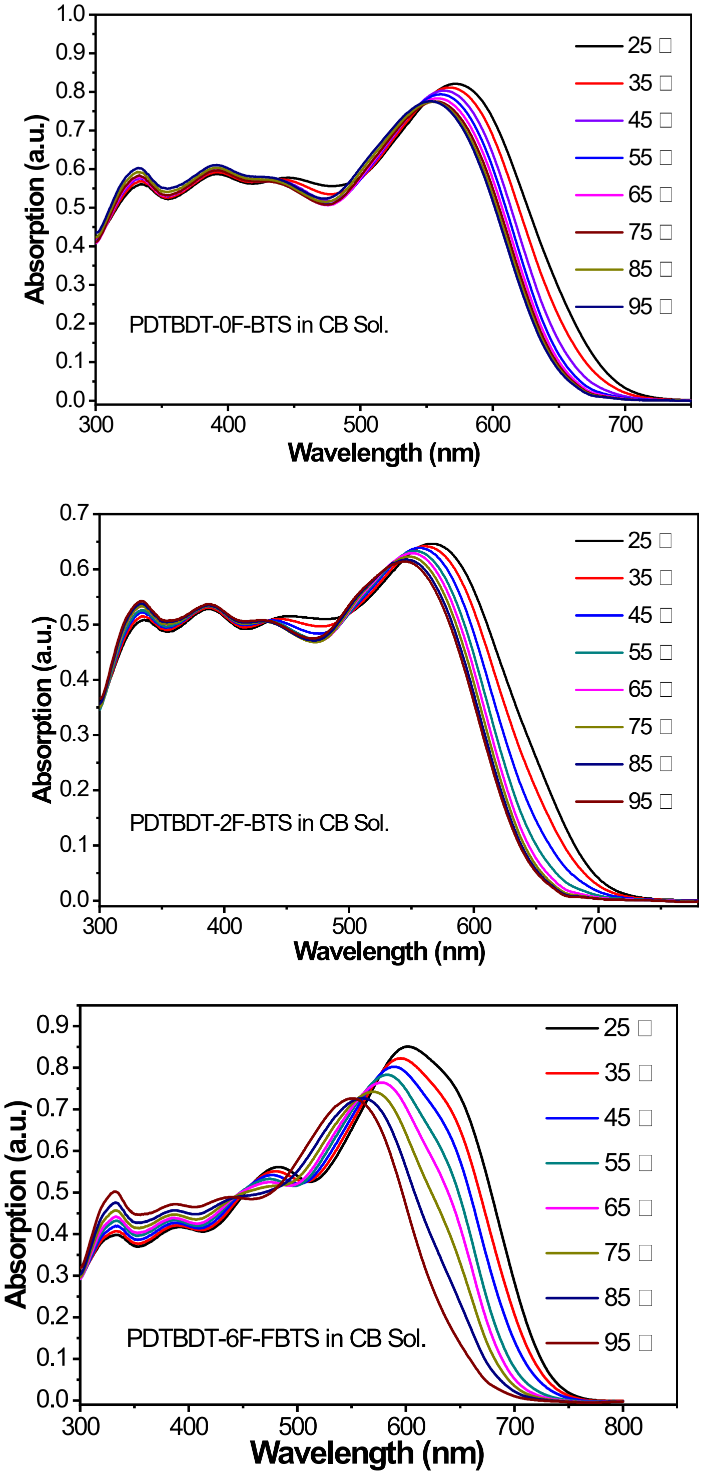

Fig. S6. TD-Abs spectra of PDTBDT-0F-BTs (a), PDTBDT-2F-BTs (b) and PDTBDT-6F-FBTs(c) in o-dichlorobenzene solution.

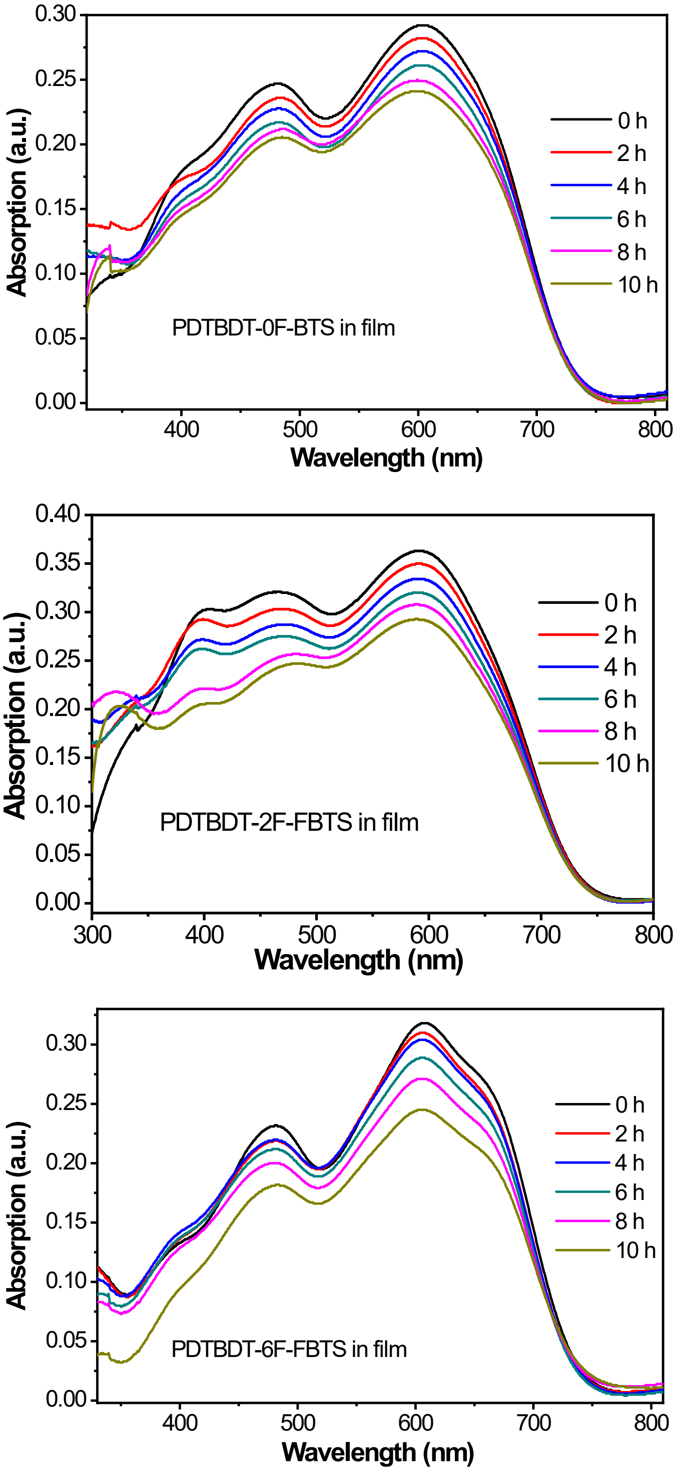

Fig. S7. Absorption spectra of PDTBDT-0F-BTs (a), PDTBDT-2F-BTs (b) and PDTBDT-6F-FBTs(c) in film

The crystal coherence lengths (CCL) of (010) and (100) peaks were determined from the Scherrer equation ($CCL = \frac{2\pi K}{FWHM}$,in which K is Scherrer constant of approximately 0.9, FWHF is full width at half maximum for scattering peak).

Table S1 Experimental data obtained from GIWAXS characterization.

Blend film	Out-of-plane (010)				In-plane (100)			
	Location ($Å^{-1}$)	d-spacing (Å)	FWHM	CCL (Å)	Location ($Å^{-1}$)	d-spacing (Å)	FWHM	CCL (Å)
PDTBDT-0F-BTS	1.47	4.27	0.565	10.33	0.28	22.44	0.21	27.43
PDTBDT-2F-BTS	1.46	4.30	0.663	8.82	0.26	24.17	0.19	30.75
PDTBDT-6F-FBTS	1.56	4.03	0.402	14.61	0.298	21.08	0.11	51.71
